# Forever green: A temporal physiologic and metabolic analysis reveals genetic drivers of the staygreen trait in maize

**DOI:** 10.1093/plcell/koaf181

**Published:** 2025-07-23

**Authors:** Christian Damian Lorenzo

**Affiliations:** Assistant Features Editor, The Plant Cell, American Society of Plant Biologists; Center for Plant Systems Biology, VIB, Gent B-9052, Belgium; Department of Plant Biotechnology and Bioinformatics, Ghent University, Gent B-9052, Belgium

Senescence—the process of growing old—and death are inevitable for all living beings, and plants are no exception. In plants, the onset and duration of senescence vary dramatically, even among varieties or accessions of a single species. Leaf senescence is a stage of plant development where cellular macromolecules are degraded and nutrients are recycled and redirected to developing organs, contributing to final seed yield (Guo et al. 2021). An important agronomic trait in crops is the staygreen (SG) phenotype, characterized by delayed senescence, prolonged photosynthesis, and increased seed yield, as well as enhanced tolerance to abiotic and biotic stresses (Antonietta et al. 2024). While the phenotype of the SG trait is easy to see, it is a much more complex process than what meets the eye (Sekhon et al. 2019). For example, some plants can present a “cosmetic type” SG appearance (Thomas and Howarth 2000), continuing to appear green although photosynthesis is arrested.

In recent research, Manwinder Brar and collaborators (2025) performed a temporal physiologic and metabolic assessment in maize to investigate important metabolites underlying the SG phenotype. The authors selected 19 inbred lines with genetically distinct lineages and different SG profiles, ranging from known SG lines to non-SG (NSG) and including previously unclassified varieties. To evaluate senescence onset and duration, the authors measured chlorophyll content, CO_2_ assimilation, photosystem II activity (Fv/Fm), and reactive oxygen species content of plants during the postflowering period. Under these parameters, all maize inbreds were clustered into 2 distinct groups: one including all SG-profiled lines and the other enriched in NSG varieties. Temporal analysis revealed that in NSG-inbred lines, Fv/Fm and chlorophyll content declined faster while reactive oxygen species increases were more pronounced as compared with SG inbreds.

Metabolome changes represent internal phenotypes that act as readouts of the plant biochemistry behind the SG trait. Therefore, researchers performed an untargeted metabolic analysis and correlated the results with the physiologic parameters measured in the SG and NSG groups. Nearly 90% of primary metabolites correlated with at least 1 phenotypic trait analyzed. Sugar alcohols, typically linked to stress mitigation, were enriched in NSG lines, a pattern consistent with accelerated senescence, whereas elevated levels of several amino acids indicated ongoing protein turnover and nitrogen remobilization. By contrast, SG lines accumulated caffeic acid, chlorogenic acid, and the flavonoids naringenin chalcone and eriodictyol—metabolites that support sustained photosynthesis. While primary metabolism showed several compounds correlating to traits, a part of the study of secondary metabolism revealed a much broader spectrum of features correlating to delayed senescence. Global analysis of specialized metabolism detected >1,000 possible compounds, which, when used as criteria to classify lines, allowed a clear distinction between SG and NSG inbreds. Metabolites such as caffeic acid, chlorogenic acid, and the flavonoids naringenin chalcone and eriodyctiol were significantly associated with higher CO_2_ assimilation and chlorophyll content.

To link metabolic changes with gene pathways, the study linked 183 metabolites and 56 candidate genes to the SG phenotype, giving geneticists and breeders a precise molecular toolkit. Researchers then built a coexpression network using publicly available gene expression datasets (Kumar et al. 2023) and searched for connections among genes coding for enzymes catalyzing the biosynthesis of the metabolites correlated to the SG trait. The analysis highlighted the presence of a large subnetwork connecting most of the putative genes and 2 additional smaller subnetworks. To functionally validate gene roles, the researchers studied mutant lines of the enzymes catalyzing naringenin chalcone and eriodictyol biosynthesis (see Figure). Single maize mutants for each gene showed an accelerated senescence as compared with their wild types. Furthermore, similar results were obtained when Arabidopsis mutants orthologs were analyzed, hinting at the conservation of the role of these metabolites. Knocking out the 2 flavonoid pathway genes hastened leaf yellowing in maize and Arabidopsis, proving their cross-species importance and spotlighting prime targets for crop improvement.

**Figure.**
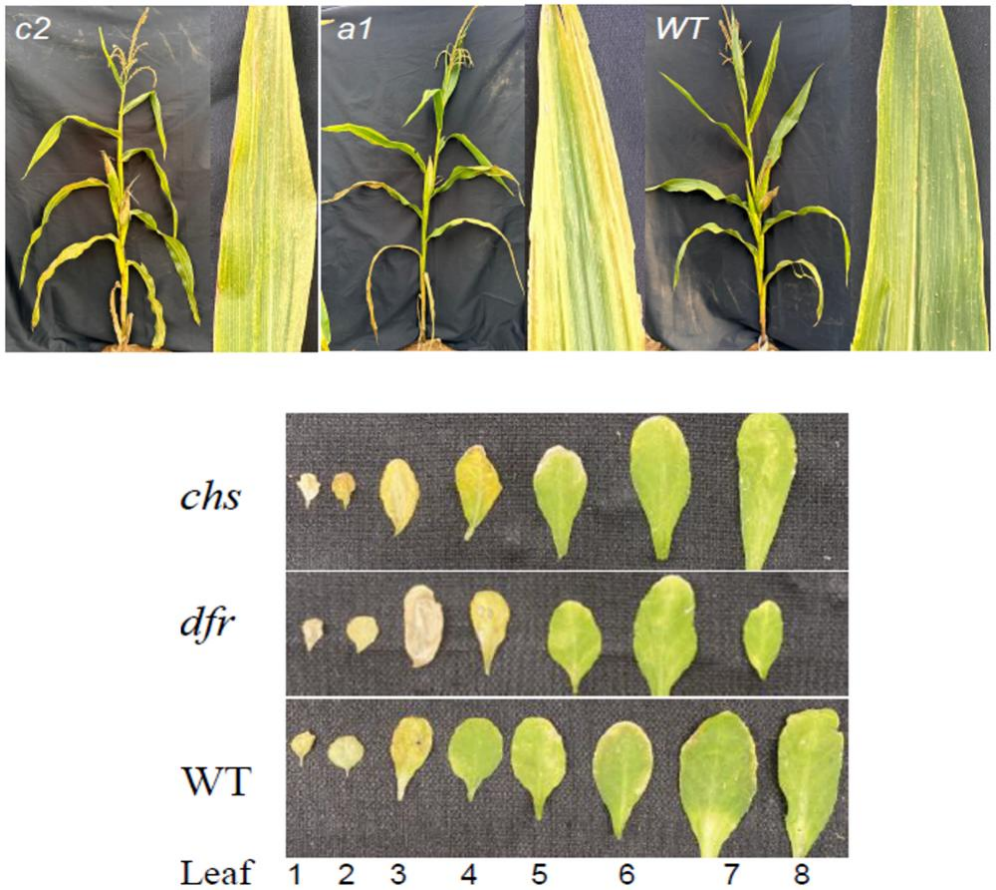
Accelerated senescence of mutants in genes catalyzing the biosynthesis of 2 important SG-related metabolites, naringenin chalcone and eriodictyol, in maize (upper panel) and their orthologs in Arabidopsis (lower panel). *Colorless2* (*c2*) and *anthocyaninless1* (*a1*) in maize are orthologs of *chalcone synthase* (*chs*) and *dihydroflavonol reductase* (*dfr*) in Arabidopsis, respectively. Adapted from Brar et al. (2025); Figures 9 and 10. WT = wild type.

In summary, this work presents a comprehensive, large-scale study on the prominent genes and metabolites involved in the SG phenotype. This time-resolved approach shows how metabolite mapping can unmask the genetics of complex crop traits, opening faster routes to greener, higher-yielding cereals.

## Recent related articles in *The Plant Cell*

- Zhang and collaborators (2025) showed the mechanism behind the kinase ATM role in Arabidopsis leaf senescence.
- Liu and collaborators (2025) performed a metabolic analysis on maize big embryo 6, revealing the role of aromatic amino acid–catalyzing enzymes in seed and plant development.

## Dive Curated Terms

The following phenotypic, genotypic, and functional terms are of significance to the work described in this paper:

ATM Gramene: AT3G48190
ATM Araport: AT3G48190

leaf senescence Planteome: TO:0000249
